# Corrosion Protection Oxide Scale Formed on Surface of Fe-Ni-M (M = Al, Cr, Cu) Inert Anode for Molten Salt Electrolysis

**DOI:** 10.3390/ma15030719

**Published:** 2022-01-18

**Authors:** Myungjae Kim, Jungshin Kang, Jiwoo Kim, Jiwoong Kim

**Affiliations:** 1 Department of Organic Materials and Fiber Engineering, Soongsil University, Seoul 06978, Korea; kmj9733@gmail.com (M.K.); jiwook725@gmail.com (J.K.); 2 Korea Institute of Geoscience and Mineral Resources, Daejeon 34132, Korea; jskang@kigam.re.kr; 3 Department of Resources Recycling, University of Science and Technology, Daejeon 34133, Korea

**Keywords:** first-principles calculation, inert anode, spinel compound, elastic property, thermal property

## Abstract

An oxide scale formed on the surface of metal anodes is crucial for determining the overall quality of molten salt electrolysis (MSE), particularly for the durability of the anode materials. However, the material properties of oxide scales are yet to be revealed, particularly in ternary spinel oxide phases. Therefore, we investigate the mechanical and thermal properties of spinel oxides via first-principles calculations. The oxides are calculated using the models of normal (cubic) and inverse (orthorhombic) spinel compounds. The d-orbital exchange correlation potential of transition metal oxides is addressed using the generalized gradient approximation plus Hubbard U. The lattice constant, formation energy, cohesive energy, elastic modulus, Poisson’s ratio, universal anisotropy index, hardness, minimal thermal conductivity, and thermal expansion coefficient are calculated. Based on the calculated mechanical and thermal properties of the spinel compound, the Fe–Ni–Al inert anode is expected to be the most suitable oxide scale for MSE applications among the materials investigated in our study.

## 1. Introduction

The carbon anodes used in the primary metal (Mg, Al, and Ti) industry inevitably result in high energy consumption, severe air pollution, and other problems [1,2]. Therefore, inert anodes are in high demand as they reduce the production of greenhouse and other harmful gases such as CO_2_, C_2_F_6_, and CF_4_ [3,4,5,6]. In addition, although the molten salt electrolysis (MSE) method is advantageous, it requires the use of expensive noble metal (Ag, Pt, Pd, and Ir) anodes [7,8,9].

Among inert anode materials, Fe–Ni-based alloy anodes are good candidates because of their excellent mechanical properties, electrical conductivity, cracking resistance, good thermal shock resistance, and ease of fabrication [10,11]. In the initial stage of electrolysis, several types of oxides are formed as scales on the surface of the anode. These oxide scales protect the alloy anode against highly corrosive conditions [12,13]. However, certain problems occur such as poor contact with molten salt, poor adhesion of the oxide scale, heterogeneous oxide scale growth, and formation of a thick oxide scale.

Therefore, studies regarding ternary inert anodes with other elements added to Fe–Ni based anodes are actively being conducted. In particular, Fe–Ni–Cr, Fe–Ni–Cu, and Fe–Ni–Al inert anodes are garnering considerable attention. In general, the corrosion of the anode surface yields oxide scales composed of binary oxides (Fe_2_O_3_, Fe_3_O_4_, NiO, Cr_2_O_3_, and Al_2_O_3_) and spinel compounds (NiFe_2_O_4_, FeAl_2_O_4_, CrFe_2_O_4_, and CuFe_2_O_4_) [14,15]. Although binary oxide scales have been extensively investigated, information regarding the thermal and mechanical properties of spinel compounds remains insufficient. Hence, in this study, we investigated spinel oxide scales, to which Al, Cr, and Cu were added.

For a normal spinel compound, the face-centered cubic (fcc) sites of the tetrahedron are occupied by 1/8 of A^2+^, and on the other side, 16/32 of the octahedral sites are occupied by B^3+^. Conversely, the inverted spinel compound occupies half of the octahedral sites, B^3+^ and A^2+^, and the other half of B^3+^ occupies tetrahedral sites [16]. The two structures can be written in the form A_X_(B_2_)*_Y_*O_4_ and B_X_(AB)*_Y_*O_4_, where *X* and *Y* represent the tetrahedral and octahedral sites of the normal and inverse spinel compounds, respectively [17]. In general, the metal ion preference for octahedral site occupation is Cu^2+^ > Cr^2+^ > Ni^2+^ > Mn^3+^ > Al^3+^ > Fe^2+^ > Co^2+^ > Fe^3+^ > Mn^2+^ [18]. This sequence shows that FeNi_2_O_4_, FeCr_2_O_4_, and FeCu_2_O_4_ are the inverse spinel phases with the molecular formula B^3+^(A^2+^B^3+^)O_4_. Therefore, AlFe_2_O_4_ is an inverse spinel phase with the molecular formula Fe^3+^(Fe^2+^Al^3+^)O_4_. All Al^3+^ cations occupy the octahedral sites, and Fe cations are distributed similarly between the octahedral and tetrahedral sites. Conversely, FeAl_2_O_4_ is a normal spinel-type structure. Based on the content of the cation (Al^3+^, and Fe^3+^) in tetrahedral sites, FeAl_2_O_4_ and AlFe_2_O_4_ are classified as normal and inverse spinel compounds, respectively [19].

The purpose of this study is to investigate the thermal and mechanical properties of normal and inverse spinel compounds that inevitably form on an alloy-based inert anode. First-principles calculations were performed to analyze the structural properties of n-NiFe_2_O_4_, n-FeAl_2_O_4_, n-CrFe_2_O_4_, and n-CuFe_2_O_4_ compounds of the normal spinel and i-NiFe_2_O_4_, i-AlFe_2_O_4_, i-CrFe_2_O_4_, and i-CuFe_2_O_4_ compounds of the inverse spinel, as well as to understand the differences in their thermal and mechanical properties. We calculated the normal spinel and inverse spinel compounds using different models. The normal spinel compound reflected a cubic (space group: *Fd-3m*) structure with formula AB_2_O_4_. In contrast, the inverse spinel compound reflected an orthorhombic (space group: *Imma*) structure, in which the pair strains were uniformly oriented relative to the cubic structure [20,21].

## 2. Computational Methods

**Figure 1:**
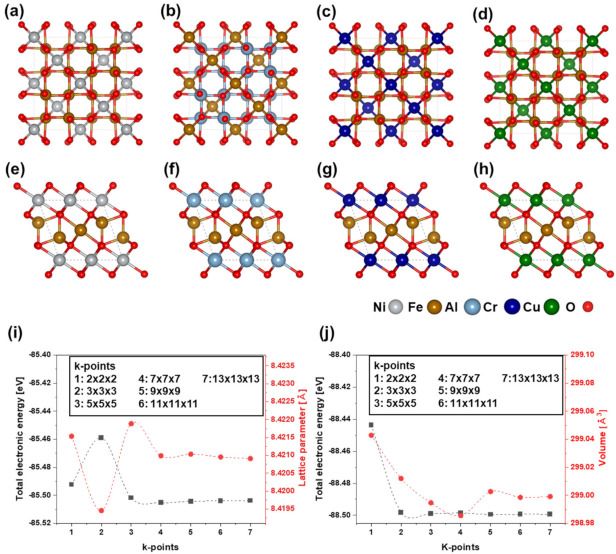
Structure models of normal (*Fd-3m*, cubic) and inverse (*Imma*, orthorhombic) spinel compound for (**a**) n-NiFe_2_O_4_, (**b**) n-FeAl_2_O_4_, (**c**) n-CrFe_2_O_4_, (**d**) n-CuFe_2_O_4_, (**e**) i-NiFe_2_O_4_, (**f**) i-AlFe_2_O_4_, (**g**) i-CrFe_2_O_4_, and (**h**) i-CuFe_2_O_4_. K-point convergence test: (**i**) variations in total electronic energy and lattice parameter for normal spinel compound model; (**j**) variations in total electronic energy and volume for inverse spinel compound model.

First-principles calculations were performed using the Vienna Ab initio Simulation Package (VASP) [22,23]. The exchange and correlation interactions were described using the generalized gradient approximation (GGA) formulated by Perdew, Burke, and Ernzerhof (PBE) [24,25]. Furthermore, we used the GGA with the Hubbard U parameterization method (GGA + U) to describe the exchange-correlation potential [26]. To integrate the Brillouin zone, we used the Monkhorst pack [27] for the normal spinel compound (cubic) of n-NiFe_2_O_4_, n-FeAl_2_O_4_, n-CrFe_2_O_4_, and n-CuFe_2_O_4_, and the inverse spinel compound (orthorhombic) of i-NiFe_2_O_4_, i-AlFe_2_O_4_, i-CrFe_2_O_4_, and i-CuFe_2_O_4_ (Figure 1a–h). An 11×11×11 k-point mesh was used for the structural optimization, total energy, and density of states calculations, whereas a 7×7×7 k-point mesh was used to calculate the elastic constants. The electronic structures were calculated using single-point energy calculations of the optimized models for normal and inverse spinel compounds. These k-points were obtained from the k-point convergence test, shown in Figure 1i,j. Accurate results were obtained using a high-energy cutoff of 500 eV with a precise energy convergence of 0.015 eV/Å. Integration was conducted using the tetrahedron method with Bloch corrections. All calculations were performed with spin-polarization. The Hubbard onsite correction term U parameters of the relevant elements for the d electrons are as follows: Fe = 4.0 eV, Ni = 6.0 eV, Cr = 3.5 eV, and Cu = 4.0 eV [28,29,30]. The elastic constants of the spinel compounds were estimated using the stress–strain method. Three different independent symmetry elements $\left(C_{11},\;C_{12},\;\mathrm{and}\;C_{44}\right)$ exist for cubic crystals. Meanwhile, orthorhombic symmetry possesses nine different independent elastic constants $(C_{11},\;C_{12},\;C_{13},\;C_{22},\;C_{23},\;C_{33},\;C_{44},C_{55},$ and $C_{66})$ [31]. The elastic properties of the ordered normal and inverse spinel compounds were calculated using the following equations [32,33]:

(1) $$B_{v}=B_{R}=1/3\left(C_{11}+2C_{12}\right)$$

(2) $$G_{v}=1/5\left(C_{11}-C_{12}+3C_{44}\right)$$

(3) $$G_{R}=5\left(C_{11}-C_{12}\right)C_{44}/4C_{44}+3\left(C_{11}-C_{12}\right)$$

(4) $$B_{v}=1/9\left(C_{11}+C_{22}+C_{33}+2C_{12}+2C_{13}+2C_{23}\right)$$

(5) $$G_{v}=1/15\left[+C_{22}+C_{33}+3C_{44}+3C_{55}+3C_{66}-(C_{12}+C_{13}+C_{23}\right)]$$

(6) $$\begin{array}{c} B_{R}=[C_{11}\left(C_{22}+C_{33}-2C_{23}\right)+C_{22}\left(C_{33}-2C_{13}\right)-2C_{33}C_{12}+C_{12}\left(2C_{23}-C_{12}\right)\\ +C_{13}\left(2C_{12}-C_{13}\right)+C_{23}){]}^{-1} \end{array}$$

(7) $$\begin{array}{l} G_{R}=15\{4[C_{11}\left(C_{22}+C_{33}-C_{23}\right)+C_{22}\left(C_{33}+C_{13}\right)+C_{33}C_{12}-C_{12}\left(C_{12}+C_{23}\right)-C_{13}(C_{13}\\ \;\;\;\;\;\;+C_{12})-C_{23}\left(C_{23}+C_{13}\right)]/C_{13}(C_{12}C_{23}-C_{13}C_{22}+C_{23}(C_{12}C_{13}\\ \;\;\;\;\;\;-C_{23}C_{11})+C_{33}{\left(C_{11}C_{22}-C_{12}^{2}+3\left(1/C_{44}+1/C_{55}+1/C_{66}\right)\right\}}^{-1} \end{array}$$

(8) $$E=9BG/\left(3B+G\right)$$

where *B*, *G*, and *E* are the bulk, shear, and Young’s moduli, respectively; *V*, *R*, and VRH represent the Voigt, Reuss, and Voigt–Reuss–Hill approximations, respectively. Additionally, the formulations employed in the present study are listed in Section 3.

## 3. Results and Discussion

The equilibrium lattice parameters, formation energies, and cohesive energies of the normal and inverse spinel compounds are listed in Table 1 [34,35,36,37,38,39]. To evaluate the structural stability, the formation energy (*E_form_*) and cohesive energy (*E_coh_*) of the spinel compounds were calculated as follows [40]:

(9) $$E_{Form}=\left[E_{tot}^{ABO}-E_{solid}^{A}\times m-E_{solid}^{B}\times n-1/2E_{gas}^{O_{2}}\times 4\right]/\left(m+n+4\right)$$

(10) $$E_{coh}=\left[E_{tot}^{ABO}-E_{atom}^{A}\times m-E_{atom}^{B}\times n-E_{atom}^{O}\times 4\right]/(m+n+4)$$

where *m* and *n* refer to the numbers of Fe, Ni, Al, Cr, Cu, and O atoms. $E_{solid}^{A}$ and $E_{solid}^{B}$ are the average energies per atom with Fe, Ni, Al, Cr, and Cu in the solid states; $E_{atom}^{A}$ and $E_{atom}^{B}$ are the energies of the Fe, Ni, Al, Cr, and Cu free atoms in the cell lattice; $E_{total}^{ABO}$ is the total energy of the A_X_(B_2_)_Y_O_4_ and B_X_(AB)_Y_O_4_ spinel compounds. It was observed that the values of the lattice parameters for all calculated stable spinel compounds were consistent with the experimental and other theoretical values. The results show that the formation energy and cohesive energy of both the normal and inverse spinel compounds were negative. The inverse spinel compound indicated a lower formation energy than the normal spinel compound. Therefore, it was expected that the inverse spinel phase would require a lower energy than the general spinel phase during oxide scale formation. In particular, the oxide scale formation rate of the i-AlFe_2_O_4_ spinel compound is expected to be high. Higher cohesive energies were observed in all normal spinel compounds, which indicated a decline in bonding strength. In addition, the lower the density of the spinel compound, the higher the possibility of peeling due to poor contact with the anode surface. Densities of normal and inverse spinel compounds were lower in the Al-containing compounds, n-FeAl_2_O_4_ and i-AlFe_2_O_4_. The n-CuFe_2_O_4_ and i-CuFe_2_O_4_ compounds were shown to have high densities.

The equilibrium bulk (*B*), shear (*G*), Young’s moduli (*E*), Poisson’s ratio (*v*), Pugh’s constant (*G*/*B*), universal anisotropy index (*A^U^*), and empirical hardness of the normal and inverse spinel compounds are listed in Table 2 [34,41]. The bulk moduli of i-NiFe_2_O_4_ and n-FeAl_2_O_4_ were larger, which indicate that they have a greater resistance to deformation than the other compounds. Similarly, the shear and Young’s moduli of i-NiFe_2_O_4_, n-FeAl_2_O_4_, and i-AlFe_2_O_4_ were higher than those of the other spinel compounds. These results show that i-NiFe_2_O_4_, n-FeAl_2_O_4_ and i-AlFe_2_O_4_ are not only more resistant to shear deformation, but can also withstand longitudinal deformation better than other spinel compounds. By contrast, the bulk, shear, and Young’s moduli values of the n-CrFe_2_O_4_, i-CrFe_2_O_4_, n-CuFe_2_O_4_, and n-CuFe_2_O_4_ compounds were lower than those of the other two compounds. In particular, n-CuFe_2_O_4_ exhibited an extremely low shear and Young’s moduli. Therefore, n-FeAl_2_O_4_ and i-AlFe_2_O_4_ are expected to exhibit excellent deformation against external stress, except for the NiFe_2_O_4_ compound oxide scale typically formed in Fe–Ni–M anodes.

The results reveal that the Poisson’s ratios of the spinel phases were between *v* = 0.25 and 0.33, indicating ionic and metallic bonding in all atoms of the spinel compound. The Poisson’s ratio is an index that describes the directionality of chemical bond; it is *v* = 0.1, for covalent bonds, *v* = 0.25 for ionic bonds, and *v* = 0.33 for metallic bonds [42]:

(11) $$v=\left(3B-2G\right)/2\left(3B+G\right)$$

In addition, the ductile/brittle behavior of spinel compounds was also considered using Pugh’s constant *G*/*B* [43]. A compound exhibits ductility when *G*/*B* is lower than 0.57; otherwise, it is brittle. The *G*/*B* value calculated for all spinel compounds was less than 0.57, indicating a high ductility; this is consistent with the Poisson’s ratio calculated previously. This indicates that all spinel compounds have high thermal shock resistance. Furthermore, it indicates that cracks progress slowly when plastic deformation occurs. Hardness is an important parameter for evaluating the wear behavior of materials [44,45]. Therefore, the hardness of the inert anode materials must be investigated. Hardness can be obtained using the Poisson’s ratio and Young’s modulus [46]. In this study, we used a relatively simple semi-empirical equation for hardness, as follows [47]:

(12) $$H_{V}=0.92k^{1.137}G^{0.708}$$

where, *G* is the shear modulus, and k is Pugh’s constant (G/B). The results are consistent with the relationship between hardness and ductility. Therefore, we concluded that the hardness values calculated in this study were reliable. In particular, n-FeAl_2_O_4_ and i-AlFe_2_O_4_ indicated the highest values of hardness, i.e., 6.22 and 6.21, respectively. As such, they were expected to exhibit excellent durability and few cracks when forming an oxide scale.

The universal anisotropy index (*A^U^*) determines the transfer probability of microcracks and the structural stability of a material [48]. A material is elastically isotropic when it satisfies *A*^U^ = 0; otherwise, it is elastically anisotropic. To characterize elastic anisotropy, we adopted the universal anisotropy index proposed for all crystal systems, as follows [49]:

(13) $$A^{U}=5G_{v}/G_{R}+B_{v}/B_{R}-6\geq 0$$

where *G* is the shear modulus; *B* is the bulk modulus; and subscripts, *V* and *R*, denote the Voigt and Reuss averages, respectively. Our calculations show that the *A^U^* values deviated from zero, indicating that both compounds were anisotropic. Furthermore, it was emphasized that these compounds were more likely to develop structural defects or microcracks during their growth into oxide scales. The ceramics should preferably be isotropic; otherwise, they will deform preferentially in a specific direction. In particular, n-CuFe_2_O_4_ exhibited a greater anisotropy than the other compounds; therefore, its oxide scale was expected to fracture easily.

**Figure 2:**
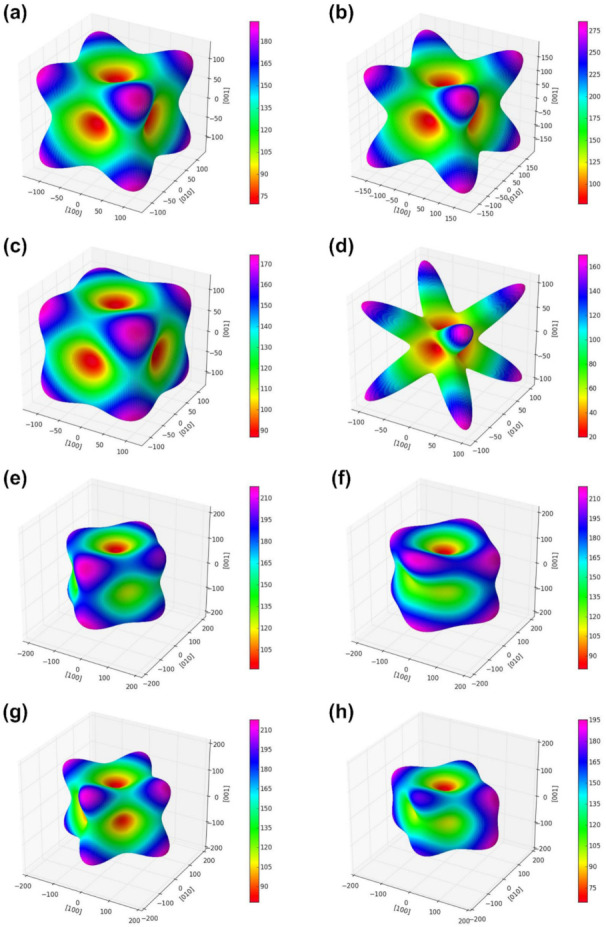
Directional dependence of Young’s modulus for spinel compounds (**a**) n-NiFe_2_O_4_, (**b**), n-FeAl_2_O_4_, (**c**) n-CrFe_2_O_4_, (**d**) n-CuFe_2_O_4_, (**e**) i-NiFe_2_O_4_, (**f**) i-AlFe_2_O_4_, (**g**) i-CrFe_2_O_4_, and (**h**) i-CuFe_2_O_4_.

Nevertheless, these factors do not contribute sufficiently to the complete description of the elastic anisotropic behavior of the crystals. The orientation dependence of the Young’s modulus is typically employed to analyze the elastic anisotropy of the crystals [50]. The elastic stress field is controlled by elastic anisotropy, which dominates the initial slip system. Therefore, the illustration of elastic anisotropy is important for predicting the stress field evolution as a function of the crystal orientation [51]. Calculating the elastic anisotropy of spinel compounds is important for understanding these properties and identifying mechanisms that will improve their durability. To further investigate the anisotropic features of the spinel compound, a three-dimensional (3D) surface representation of the elastic anisotropy of the crystal was created. For cubic and orthorhombic crystals, the Young’s modulus in any orientation is expressed as [52,53]:

(14) $$1/E=S_{11}-2(S_{11}-S_{12}-S_{44}/2)\left(l_{1}^{2}l_{2}^{2}+l_{2}^{2}l_{3}^{2}+l_{1}^{2}l_{3}^{2}\right)$$

(15) $$1/E=S_{11}l_{1}^{4}+\left(2S_{12}+S_{66}\right)l_{1}^{2}l_{2}^{2}+S_{22}l_{1}^{4}+\left(2S_{23}+S_{44}\right)l_{2}^{2}l_{3}^{2}+S_{33}l_{3}^{4}\;+\left(2S_{13}+S_{55}\right)l_{1}^{2}l_{3}^{2}$$

where *l*_1_, *l*_2_, and *l*_3_ are direction cosines with respect to the a, b, and c directions of the lattice, respectively. The spatial 3D surface representation of the Young’s modulus is shown in Figure 2a–h. Young’s modulus surfaces are perfectly spherical for isotropic crystals, but not for normal and inverse spinel compounds. This shows that the normal and inverse spinels exhibit elastic anisotropy. Furthermore, the Young’s moduli of the normal and inverse spinel compounds in the normal direction of three low-index crystal planes {100}, {110}, and {111} were calculated. The equations for normal directions of the planes are as follows [51]:

(16) $$1/E_{hkl}=S_{11}-2S_{0}[{\left(hk\right)}^{2}+{\left(hl\right)}^{2}+{\left(lk\right)}^{2}/{\left(h^{2}+k^{2}+l^{2}\right)}^{2}$$

(17) $$S_{0}=S_{11}-S_{12}-1/2S_{44}$$

The corresponding calculation results are listed in Table 3. The calculated normal and inverse spinel compounds showed different Young’s moduli values depending on the plane direction. The general spinel compounds indicated the highest stiffness in the {111} direction, regardless of the composition. The inverse spinel compounds indicated the highest stiffness in the {100} direction, regardless of the composition. In terms of n-CuFe_2_O_4_, the Young’s moduli differed significantly depending on the plane direction. The calculated minimum and maximum values of the Young’s modulus were 19.07 and 171.6 GPa for the {100} plane: 47.87 and 144.33 GPa for the {110} plane, and 54.57 and 278.4 GPa for the {111} plane. In general, spinel compounds with a high rigidity along the plane direction were indicated as n-NiFe_2_O_4_ in {100}, n-FeAl_2_O_4_ in {110}, and n-CuFe_2_O_4_ in {111}.

Generally, the Debye temperature (*θ_D_*) is a fundamental parameter associated with a number of physical properties of materials, including their elastic constants, specific heat, chemical bonding, and melting point [54,55]. Excitation due to low-temperature vibrations occurs only in acoustic vibrations. The following equation was used to estimate the magnitude of the *θ_D_* at the average speed of sound [55]:

(18) $$\theta_{D}=h/k_{B}{\left[3n/4\pi \left(N_{A}\rho /M\right)\right]}^{1/3}v_{m}$$

where *M* is the mean molecular weight, *n* the total number of atoms in the formula unit, *ρ* the mass density, *h* the Plank constant, *k_B_* the Boltzmann constant, and *N_A_* the Avogadro number. For polycrystalline materials, the average velocity of sound $v_{m}$ is expressed as [9]:

(19) $$v_{m}={\left[1/3\left(1/v_{l}^{3}+2/v_{t}^{3}\right)\right]}^{-1/3}$$

(20) $$v_{l}=\sqrt{\left(B+\left(4/3\right)G\right)/\rho }$$

(21) $$\nu_{t}=\sqrt{G/\rho }$$

where *v_l_* and *v_t_* represent the longitudinal and transverse sound velocities in anisotropic materials, respectively. They can be determined in terms of the bulk modulus *B* and shear modulus *G* [56]. For crystal structures, *θ_D_* defines the highest temperature of the material for the normal vibrational mode, specific heat, and melting temperature. As shown in Table 4, the n-FeAl_2_O_4_ and i-AlFe_2_O_4_ spinels had the largest *θ_D_* values of 617.7 K and 733.2 K, respectively. The calculated average sound velocities of these n-FeAl_2_O_4_ and i-AlFe_2_O_4_ compounds were relatively large, because they had large elastic moduli and small densities. The *v_l_* and *v_t_* values are associated with the density, shear modulus, and bulk modulus. The high volume leads to the formation of thick spinel oxide scale, which reduces the contact with the electrolyte, thereby reducing the electrochemical properties. In contrast, when thin spinel oxide scales are formed, the electrochemical properties are increased but the mechanical behavior is decreased. It is expected that normal spinel oxide scales are bulkier than inverse spinel oxide scales and form thick oxide scales, whereas inverse spinel oxide scales are expected to form thin oxide scales. When comparing normal and inverse spinel of the same element, n-FeAl_2_O_4_ and i-AlFe_2_O_4_ compounds are expected to form the most ideal oxide scale. However, no experimental or theoretical evidence for comparing our results in terms of density, *θ_D_*, and elastic wave is available in the literature.

**Figure 3:**
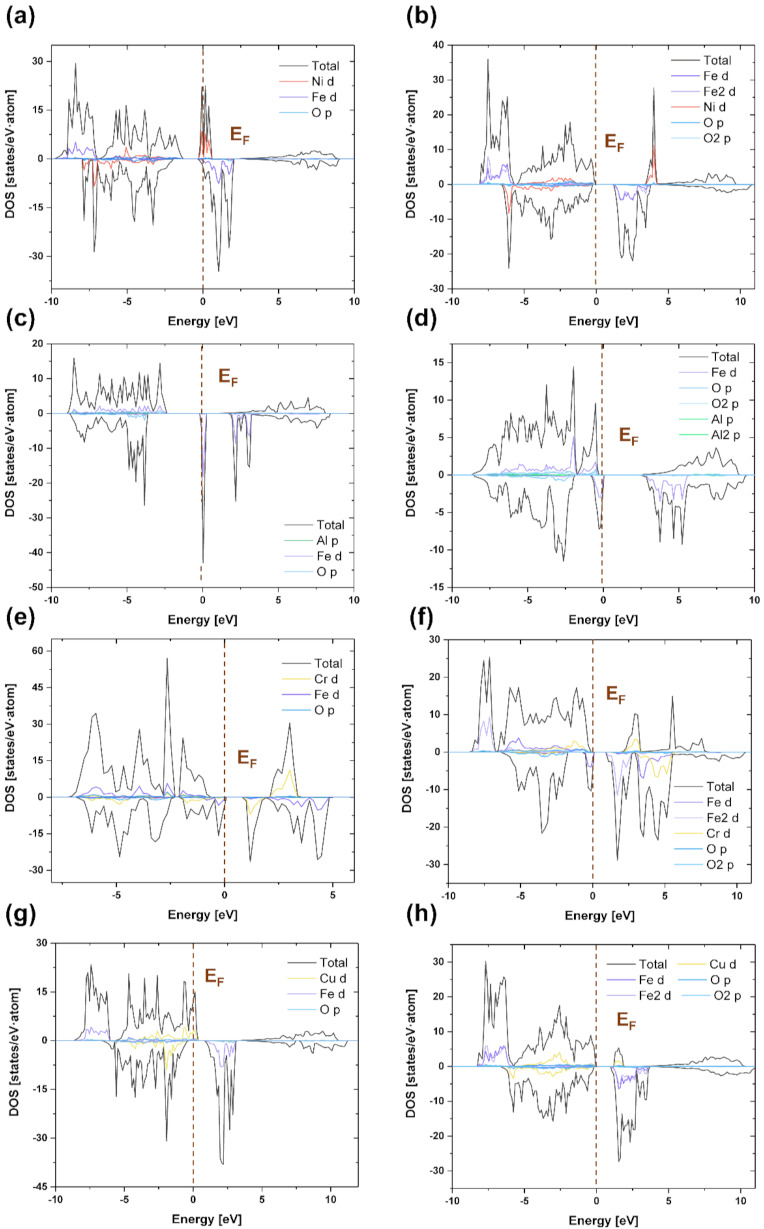
TDOS and PDOS of normal and inverse spinel compounds (**a**) n-NiFe_2_O_4_, (**b**) i-NiFe_2_O_4_, (**c**) n-FeAl_2_O_4_, (**d**) i-AlFe_2_O_4_, (**e**) n-CrFe_2_O_4_, (**f**) i-CrFe_2_O_4_, (**g**) n-CuFe_2_O_4_, and (**h**) i-CuFe_2_O_4_. The vertical dashed lines indicate the Fermi level.

To investigate the causes of the mechanical and electrochemical properties of normal and inverse spinel compounds, we calculated and compared their electronic structures. Figure 3 shows the calculated total density of states (TDOS) and partial density of states (PDOS) of (a) n-Fe_2_NiO_4_, (b) i-Fe_2_NiO_4_, (c) n-Fe_2_AlO_4_, (d) i-AlFe_2_O_4_, (e) n-CrFe_2_O_4_, (f) i-CrFe_2_O_4_, (g) n-CuFe_2_O_4_, and (h) i-CuFe_2_O_4_ spinel compounds. The energy level 0 eV on the x-axis represents the Fermi level of the spinel compound. Figure 3a,b shows that Fe-3d orbital is separated into t_2g_-up and e_2g_-up to generate a valence band using O-2p orbital and conduction in Fe-3d orbital [57]. Figure 3c,d shows that the high peaks near the Fermi level correspond to Fe 3d states. In addition, in the Valence band, Fe-3d and Al-3p states contribute to the formation of the highest peak. The high peak in the conduction band is caused by the Fe-3d states [58]. As shown in the figure, majority spins were observed in the valence band and minority spins in the conduction band. Figure 3e,f show shifts in the Fermi level of the 3d state peaks of Fe and Cr cations between the two spin directions for this compound. As a result, the 3d states of the Fe and Cr transition metals were confirmed as the possible cause of magnetism. The highest peak in the valence band is due to Fe-3d states. The highest peak in the conduction band is caused by Fe-3d states. In particular, it was observed that the Fe-3d and Cr-3d states overlapped in the peak with a high electron density. Figure 3g,h shows that the peak at the Fermi level was observed to be connected with the Cr-3d state. The highest peak in the valence band is caused by the Fe-3d and Cr-3d states, and the conduction band is caused by the Fe-3d states. On the other hand, in the case of i-CuFe_2_O_4_, there was no large peak near the Fermi level. In particular, minority Cr-3d states were observed in the conduction band. As a result, it was observed that the transition metal elements were determined by 3d states, and Al and O elements were determined by 2p states.

The spinel oxide scales i-NiFe_2_O_4_, i-AlFe_2_O_4_, n-CrFe_2_O_4_, i-CrFe_2_O_4_, and i-CuFe_2_O_4_ exhibit insulator properties. In contrast, n-NiFe_2_O_4_, n-FeAl_2_O_4_, and n-CuFe_2_O_4_ exhibit conductor properties. As many densities of states near the Fermi level contribute to charge storage, the n-NiFe_2_O_4_, n-FeAl_2_O_4_, and n-CuFe_2_O_4_ spinel compounds should improve their electrochemical performance after they are formed in the scale [41]. In particular, the TDOS of NiFe_2_O_4_ shows a strong peak in the spin-down state close to the Fermi energy level (±0.2 eV). Al-O bonds are ionic in nature. As Al atoms are replaced by Fe atoms, the charge density increases, forming Fe-O bonds with both ionicity and covalent properties [59]. Therefore, it is expected that the electrical conductivity properties of the n-NiFe_2_O_4_ and n-FeAl_2_O_4_ oxide scales will be excellent.

**Figure 4:**
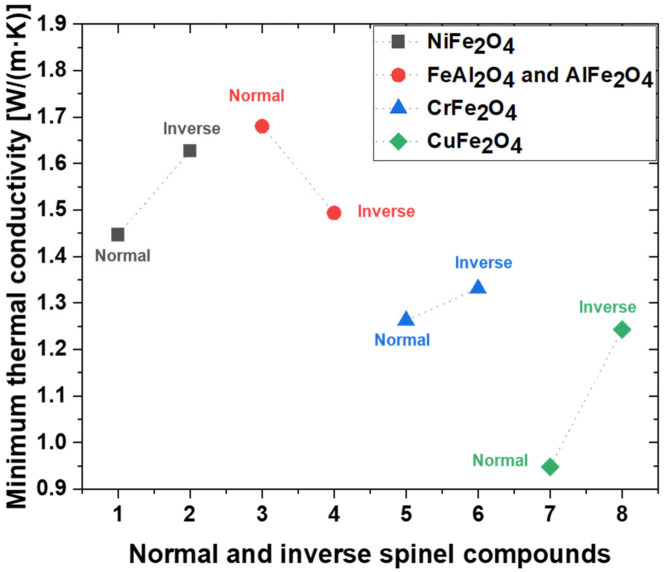
Minimum thermal conductivity (*K_min_*) of normal and inverse spinel compound.

The minimum thermal conductivities of the spinel compounds are shown in Figure 4. Thermal conductivity describes the diffusivity of heat flow via phonon transport in a temperature gradient. Crystalline materials typically exhibit four distinct regions in the thermal conductivity–temperature curve. To investigate the behavior at high temperatures, the last of the four areas must be emphasized, i.e., the high-temperature region that exceeds *θ_D_*, where the thermal conductivity exhibits a minimum value. The minimum thermal conductivity was calculated using Clarke’s model, which can be expressed as [60,61]:

(22) $$k_{min}=0.87k_{B}{\bar{M_{a}}}^{-2/3}E^{1/2}\rho^{1/6}),\;{\bar{M}}_{a}=\left[M/\left(m\cdot N_{A}\right)\right]$$

where *K_B_* is the Boltzmann constant, *ρ* is the density, *E* is the Young’s modulus, $N_{A}$ is the Avogadro number, *M* is the molar mass, *m* is the total number of atoms per formula, and ${\bar{M}}_{a}$ is the average mass per atom. The thermal conductivity of solid materials varies with temperature and pressure because the phonon means that the free paths and vibration properties of the material depend on the temperature and pressure [62]. At high temperatures, solid materials converge to the minimum value of thermal conductivity, as suggested by Clarke. The minimum thermal conductivity is proportional to the mean acoustic velocity, and this velocity is affected by the rigidity of the material. Therefore, the results of this study show the minimum thermal conductivity in the order n-CuFe_2_O_4_ > n-CrFe_2_O_4_ > i-CuFe_2_O_4_ > i-CrFe_2_O_4_ > n-NiFe_2_O_4_ > i-FeAl_2_O_4_ > i-NiFe_2_O_4_ > n-FeAl_2_O_4_. According to the obtained mechanical properties and thermal conductivity, n-FeAl_2_O_4_ and i-AlFe_2_O_4_ exhibited mechanical stability and a low thermal conductivity. It is beneficial to provide the physical property information when n-FeAl_2_O_4_ and i-AlFe_2_O_4_ are regarded as potential candidates for inert anode oxide scales.

**Figure 5:**
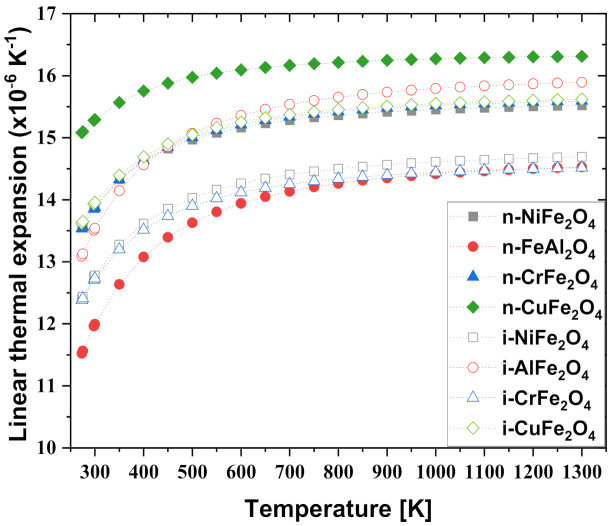
Comparison of calculated thermal expansion coefficients of normal and inverse spinel compound.

Figure 5 shows the measured thermal expansion coefficients of the normal and inverse spinel compounds. The coefficient of thermal expansion is an important factor that affects non-uniform epitaxial growth. This is because the significant difference in the coefficient of thermal expansion between dissimilar materials results in defects such as cracks and mismatch dislocations at the boundary [63,64]. The effect of temperature was analyzed using the Debye–Grüneisen model [65,66]. Investigations of other NiFe_2_O_4_ spinel compounds indicated comparable values between 11–18 × 10^−6^ K^−1^ [67]. This value is consistent with the experimental data within 10% between 275 and 400 K, but significantly (15–16%) overestimates the measured expansion above this temperature. The possible reason for the difference is that the anharmonicity correction of the Debye–Grüneisen model is too large to fit the experimental values. In addition, several different ferrite spinels with NiFe_2_O_4_ were investigated in other studies, all of which indicate expansion at rates of 7–13 × 10^−6^ K^−1^ at 1000 K [68]. This indicates that all of the normal and inverse spinel compounds investigated were overestimated. This deviation is expected because the calculation was performed on a perfect crystal, whereas the measured values are dependent on the purity of the sample, in which impurities, defects, and grain boundaries may be present.

## 4. Conclusions

We investigated the mechanical and thermal properties of normal and inverse spinel (NiFe_2_O_4_, FeAl_2_O_4_, AlFe_2_O_4_, CrFe_2_O_4_, and CuFe_2_O_4_) compounds using first-principles calculations via the GGA + U approach. In particular, n-CuFe_2_O_4_ demonstrated the lowest compression resistance and theoretical hardness, whereas n-FeAl_2_O_4_ exhibited the highest compression resistance and theoretical hardness. The trend of *θ_D_* was similar to the trends of shear and Young’s moduli. Meanwhile, the trends for the mechanical and thermophysical properties correspond to the Poisson’s ratio and Pugh’s constants of our solid solution candidates. Mechanical anisotropy was described by both the universal anisotropy index and spatial 3D surfaces. The minimum thermal conductivity of n-FeAl_2_O_4_ was the highest at 1.680 W/(m∙K), whereas that of n-CuFe_2_O_4_ was the lowest at 0.948 W/(m∙K). According to our calculations, NiFe_2_O_4_, FeAl_2_O_4_, and AlFe_2_O_4_ are expected to exhibit excellent thermal shock resistance and anode surface adhesion. Therefore, good performances can be expected when an inert Fe–Ni–Al anode is used in the MSE process. We hope that this study will provide some insights for the further investigation of spinel compound oxide scales and the design of Fe–Ni-based inert anodes for electrolysis.

## Figures and Tables

**Table 1:** Calculated lattice parameters (*a*_0_, *b*_0_, and *c*_0_ in Å), formation energy (*E_form_* in eV/atom), cohesive energy (*E_coh_* in eV), and volume (*V*_0_ in Å^3^). For comparison, previous experimental and other theoretical values are listed.

Compound	Spinel Type	*a*_0_	*b*_0_	*c*_0_	*E_form_*	*E_coh_*	*V*_0_
NiFe_2_O_4_	Normal	8.420	8.420	8.420	−0.951	−1.291	597.01
		8.426 ^a^	8.426 ^a^	8.426 ^a^	-	-	-
		8.339 ^b^	8.339 ^b^	8.339 ^b^	-	-	-
NiFe_2_O_4_	Inverse	5.937	5.982	8.417	−2.008	−2.691	298.98
FeAl_2_O_4_	Normal	8.226	8.226	8.226	−1.334	−1.624	556.77
		8.230 ^c^	8.230 ^c^	8.230 ^c^	-	-	-
		8.119 ^d^	8.119 ^d^	8.119 ^d^	-	-	-
AlFe_2_O_4_	Inverse	5.838	5.912	8.497	−2.575	−3.154	301.41
CrFe_2_O_4_	Normal	8.510	8.510	8.510	−1.119	−1.441	614.21
CrFe_2_O_4_	Inverse	6.019	5.993	8.678	−2.328	−2.971	313.10
CuFe_2_O_4_	Normal	8.466	8.466	8.466	−0.963	−1.274	606.76
		8.465 ^e^	8.465 ^e^	8.465 ^e^	-	-	-
		8.367 ^f^	8.367 ^f^	8.367 ^f^	-	-	-
CuFe_2_O_4_	Inverse	5.829	5.907	8.846	−1.936	−2.559	304.66

^a^ Ref. [34] calculation data. ^b^ Ref. [35] experimental data. ^c^ Ref. [36] calculation data. ^d^ Ref. [37] experimental data. ^e^ Ref. [38] calculation data. ^f^ Ref. [39] experimental data.

**Table 2:** Calculated mechanical properties of two types of spinel compounds: bulk modulus (*B* in GPa), shear modulus (*G* in GPa), Young’s modulus (*E* in GPa), Poisson’s ratio (*v*), Pugh’s constant (*G*/*B*), universal anisotropy index (*A^U^*), and empirical hardness (*H_v_* in GPa) for NiFe_2_O_4_ and FeAl_2_O_4_ with normal and inverse spinel compounds.

Compound	Spinel Type	*B*	*G*	*E*	*v*	*G*/*B*	*H_V_*	*A^U^*
NiFe_2_O_4_	normal	161.41	47.74	130.15	0.36	0.29	3.91	1.64
		177.1 ^a^	-	-	-	-	-	-
NiFe_2_O_4_	inverse	170.69	61.54	164.76	0.34	0.36	5.57	0.69
FeAl_2_O_4_	normal	184.79	64.93	173.51	0.34	0.35	6.22	2.98
		172.4 ^b^	56.9 ^b^	153.8 ^b^	-	-	-	-
AlFe_2_O_4_	inverse	159.64	61.86	164.19	0.32	0.38	6.21	1.11
CrFe_2_O_4_	normal	155.03	48.88	132.63	0.35	0.31	4.07	0.73
CrFe_2_O_4_	inverse	164.74	55.03	148.33	0.35	0.33	4.92	1.49
CuFe_2_O_4_	normal	152.78	27.89	77.82	0.41	0.18	1.85	9.25
CuFe_2_O_4_	inverse	156.69	49.72	134.81	0.35	0.31	4.23	1.11

^a^ Ref. [34] calculation. ^b^ Ref. [40] calculation.

**Table 3:** Calculated values of Young’s modulus (E_hkl_ in GPa) along normal directions of planes {100}, {110}, and {111}.

Compound	Spinel Type	{100}	{110}	{111}
NiFe_2_O_4_	normal	61.06	118.53	148.19
NiFe_2_O_4_	inverse	171.60	57.58	65.50
FeAl_2_O_4_	normal	67.94	144.33	200.82
AlFe_2_O_4_	inverse	159.69	55.72	68.00
CrFe_2_O_4_	normal	73.22	111.76	129.65
CrFe_2_O_4_	inverse	164.75	47.87	62.20
CuFe_2_O_4_	normal	19.07	109.96	278.40
CuFe_2_O_4_	inverse	158.94	44.86	54.57

**Table 4:** Calculated thermal properties: density (*ρ* in g/cm^3^), transverse (*v_t_* in m/s), longitudinal (*v_l_* in m/s), mean speed of sound (*v_m_* in m/s), and Debye temperature (*θ_D_* in *K*).

Compound	Spinel Type	*ρ*	*v_t_*	*v_l_*	*V_m_*	*θ_D_*
NiFe_2_O_4_	normal	5.215	3067	6659	3456	463.2
NiFe_2_O_4_	inverse	5.207	3477	7047	3904	523.8
FeAl_2_O_4_	normal	4.147	4003	8183	4496	617.7
AlFe_2_O_4_	inverse	4.466	3725	7370	4176	562.8
CrFe_2_O_4_	normal	4.925	3205	6802	3607	477.8
CrFe_2_O_4_	inverse	4.830	3418	7109	3842	507.3
CuFe_2_O_4_	normal	5.238	2329	6079	2641	353.1
CuFe_2_O_4_	inverse	5.216	3134	6638	3527	469.1

## Data Availability

Data sharing is not applicable.
